# Aneurysm Severity is Increased by Combined Mmp-7 Deletion and N-cadherin Mimetic (EC4-Fc) Over-Expression

**DOI:** 10.1038/s41598-017-17700-8

**Published:** 2017-12-11

**Authors:** Cressida A. Lyon, Helen Williams, Rosaria Bianco, Steven J. Simmonds, Bethan A. Brown, Kerry S. Wadey, Frank C. T. Smith, Jason L. Johnson, Sarah J. George

**Affiliations:** Bristol Medical School, Level 7, Bristol Royal Infirmary, BRISTOL, UK

## Abstract

There is an unmet need for treatments to reduce abdominal aortic aneurysm (AAA) progression. Vascular smooth muscle cell (VSMC) apoptosis precipitates AAA formation, whereas VSMC proliferation repairs the vessel wall. We previously demonstrated that over-expression of EC4-Fc (truncated N-cadherin), or deletion of matrix-metalloproteinase-7 (Mmp-7) reduced VSMC apoptosis in mouse atherosclerotic plaques. Additionally, MMP-7 promotes VSMC apoptosis by cleavage of N-cadherin. We investigated their combined effect on AAA formation. Increased apoptosis and proliferation were observed in human AAA (HAAA) sections compared to normal aortae (HA). This coincided with increased MMP-7 activity and reduced N-cadherin protein levels in HAAA sections compared to HA. Using a mouse model of aneurysm formation, we showed that the combination of Mmp-7 deletion and EC4-Fc overexpression significantly increased AAA severity. Medial apoptosis and proliferation were both significantly reduced in these mice compared to control mice. *In vitro*, MMP-7 inhibition and EC4-Fc administration significantly supressed human aortic VSMC apoptosis (via activation of PI-3 kinase/Akt signalling) and proliferation. In conclusion, combined Mmp-7 deletion and systemic over-expression of EC4-Fc reduced both proliferation and apoptosis. Reduced proliferation-mediated repair over-rides any benefit of reduced apoptosis, increasing aneurysm severity. Future studies should therefore focus on retarding VSMC apoptosis whilst promoting VSMC proliferation.

## Introduction

Abdominal aortic aneurysms (AAA) are a common and significant cause of premature death in the UK. It is estimated to be the tenth most common cause of mortality and accounts for 2% of all deaths. Difficulty in diagnosing potentially lethal aneurysms prior to rupture has led to screening of men over 65 years old for aortic aneurysms using ultrasound in the UK. If a large aneurysm of 5.5 cm or larger is diagnosed, surgical intervention is recommended. However, if the patient is diagnosed with a smaller aneurysm, there is no specific treatment. These patients have six monthly scans to monitor the progression of the aneurysm. Approximately 70% of these patients require treatment within 5 years due to increased AAA severity^1^. Therefore, a drug treatment to reduce aneurysm progression and rupture would be a major breakthrough.

Aneurysms are characterised by localised structural deterioration of the artery wall, thinning of the medial layer and degeneration of the internal elastic lamina. Loss of medial vascular smooth muscle cells (VSMCs) is an important contributor to this disease as the VSMCs provide tensile strength to the vessel wall as well as producing extracellular matrix. Various lines of evidence suggest that VSMCs in the artery wall undergo apoptosis during the development of an aneurysm. Medial VSMC density was significantly reduced, and apoptosis was significantly increased in aneurysmal compared to normal human abdominal aorta tissue specimens^2^. Additionally, p53, a mediator of programmed cell death was increased in these diseased vessels^2^, as were perforin and Fas which are also involved in apoptosis^3^. Data from animal models of aneurysm formation corroborates the contribution of apoptosis in AAA^4,5^. Increased apoptosis (detected by TUNEL, caspase-3 activity and histone-associated DNA fragmentation) was observed in angiotensin-II (Ang-II) induced aneurysms in the apolipoprotein E null (Apoe^−/−^) mouse model^5,6^.

It is feasible that promoting VSMC survival may reduce aneurysm formation and progression and have potential as a therapeutic approach. To date only one direct study has assessed the effect of inhibiting apoptosis on aneurysm formation and progression^6^. Yamanouchi and colleagues demonstrated that a broad spectrum caspase inhibitor significantly reduced aneurysm incidence and size in Ang-II infused Apoe^−/−^ mice^6^. Additionally, various studies have shown a link between reduced aneurysm formation and attenuated apoptosis and highlighted the use of anti-apoptotic strategies for the treatment of aneurysms^5,7–9^. Although there is clear potential for anti-apoptotic strategies to reduce aneurysm formation and progression, clinically useful compounds have not yet been identified. VSMC proliferation may also play a major role in the pathobiology of AAA. VSMC proliferation is a vitally important mechanism of repairing the damaged vessel wall and has been found to become activated after vessel wall dilation and degeneration of the elastic laminae^10^.

We have identified two strategies that reduce VSMC apoptosis in mouse. Firstly, via the cell-cell adhesion protein N-cadherin. The cadherins are a superfamily of transmembrane glycoproteins that mediate homophilic, Ca^2+^-dependent cell-cell contact^11–13^. We have previously shown that soluble N-cadherin (SNC), and a smaller truncation of SNC, called EC4-Fc, significantly increase features of atherosclerotic plaque stability^14,15^. These studies also showed that SNC-Fc and EC4-Fc are able to modulate VSMC proliferation, migration and apoptosis^14,15^.

The second strategy is inhibition of matrix metalloproteinase-7 (MMP-7). Our interest in MMP-7 was first piqued by our comparison of the divergent effects of various MMPs on atherosclerotic plaque stability, which found that Mmp-7/Apoe double knockout mice had a higher VSMC content, suggestive of increased plaque stability^16^. Our group have since shown that MMP-7 promotes VSMC apoptosis by cleavage of N-cadherin and may therefore modulate atherosclerotic plaque development and rupture^17^. We observed that mouse VSMCs deficient in MMP-7 were 44% less susceptible to Fas-L induced apoptosis than wild-type VSMCs^17^. Additionally, broad-spectrum MMP inhibition is known to reduce proliferation by limiting N-cadherin shedding from the cell surface^18^.

The aims of this study were firstly, to investigate the relevance of our target pathways and molecules in human AAA (HAAA) samples, secondly to investigate the effects of EC4-Fc over-expression and Mmp-7 deletion on AAA development and progression, using the mouse Ang-II induced aneurysm model and thirdly, to study the effects of EC4 administration and MMP7 inhibition *in vitro*.

## Results

### Enhanced apoptosis, proliferation and MMP-7 activity and reduced N-cadherin levels were observed in human AAA samples

**Figure 1:**
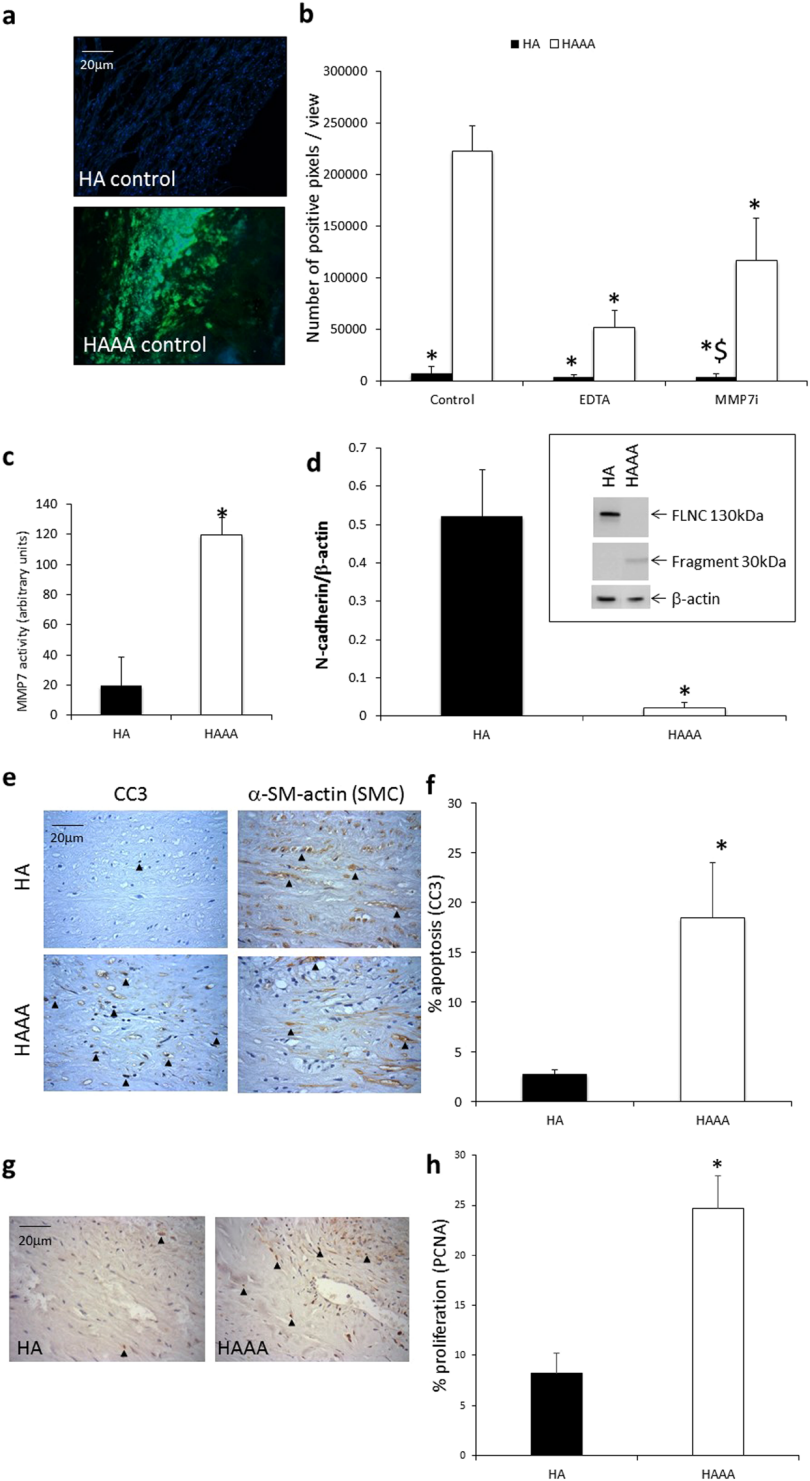
Enhanced apoptosis, proliferation and MMP-7 activity and reduced N-cadherin levels were observed in human AAA samples. (**a**) Representative images of *in situ* zymography. Green fluorescence indicates gelatinolytic activity. Nuclei are blue (DAPI). Scale bar indicates 20 µm. (**b**) Analysis of *in situ* zymography, represented as the number of green pixels/view to show gelatinolytic activity. EDTA: broad spectrum MMP inhibitor. * indicates a significant difference from HAAA control, ^$^ indicates a significant difference from HAAA + MMP-7 inhibitor (MMP-7i) (mean + SEM, n = 4, p < 0.05, ANOVA, Student Newman Keuls Post test). (**c**) MMP-7 fluorescent activity assay of tissue lysates from HAAA and HA. * indicates a significant difference from HA (mean + SEM, n = 9, p < 0.05, Mann Whitney test). (**d**) Western blot analysis for N-cadherin and loading control (β-actin) in tissue lysates from HAAA and HA. Graph shows full length N-cadherin data. * indicates a significant difference from HA (mean + SEM, n = 9, p < 0.05, Mann Whitney test). Image shows a representative blot. The full length Western blot is included as Supplementary Figure 1. (**e**) Representative images of cleaved caspase-3 and α-SMC-actin immunohistochemistry. Positive cells are brown, negative nuclei are blue. Arrows indicate some positive cells. Scale bar indicates 20 µm. (**f**) Analysis of cleaved caspase-3 immunohistochemistry. Apoptosis was measured by counting the proportion of positive cells. * indicates a significant difference from HA (mean + SEM, n = 8, p < 0.05, t test). (**g**) Representative images of PCNA immunohistochemistry. Positive cells are brown, negative nuclei are blue. Arrows indicate some positive cells. Scale bar indicates 20 µm. (**h**) Analysis of PCNA immunohistochemistry. Proliferation was measured by counting the proportion of positive cells. * indicates a significant difference from HA (mean + SEM, n = 8, p < 0.05, Students t test).

MMP-7 activity was analysed *by in situ* zymography. Significantly higher levels of gelatinolytic activity were detected in human AAA (HAAA) samples compared to control human healthy aorta (HA) tissues (Fig. 1a,b). When EDTA (a broad spectrum MMP inhibitor) was added, gelatinolytic activity was significantly reduced (Fig. 1b), suggesting that the majority of the proteolytic activity was due to MMPs. A MMP-7 selective inhibitor also significantly reduced gelatinolytic activity (Fig. 1b), suggesting that the observed activity in HAAA tissue was at least in part due to MMP-7. A fluorescent MMP-7 activity assay confirmed these findings, demonstrating significantly elevated MMP-7 activity in HAAA biopsies compared to HA (Fig. 1c). In line with the proposition that MMP-7 cleaves N-cadherin, western blot analysis of tissue lysates showed that protein levels of full length N-cadherin were significantly lower in HAAA compared to HA, whilst more N-cadherin fragment was observed in the HAAA samples (Fig. 1d).

Concomitantly, apoptosis, measured by cleaved caspase-3 immunohistochemistry, was significantly higher in the HAAA samples compared to the HA samples (Fig. 1e,f). Serial sections subjected to immunohistochemistry for α-smooth muscle actin, revealed apoptosis occurred in smooth muscle cell rich areas of the aneurysm wall (Fig. 1e). Proliferation, assessed by PCNA immunohistochemistry, was also significantly higher in the HAAA samples compared to the HA samples (Fig. 1g,h), perhaps suggestive of repair processes occurring in the aneurysmal samples.

### Ang-II induced aneurysm severity in Apoe^−/−^ mice was increased by combined Mmp-7 knockout and EC4-Fc over-expression

As expected and in line with our previous study^15^, the plasma concentration of EC4-Fc was increased in mice transfected with HDAd EC4-Fc compared with that of HDAd-Fc transfected control mice (data not shown). Importantly, low density lipoprotein levels were comparable between the four treatment groups (Mmp-7^+/+^ Fc: 22 ± 7 mM, Mmp-7^+/+^ EC4-Fc: 22 ± 6 mM, Mmp-7^−/−^, Fc: 20 ± 7 mM, Mmp-7^−/−^ EC4-Fc: 23 ± 4 mM). Additionally, no adverse effects on mouse behaviour or appearance were observed.

**Figure 2:**
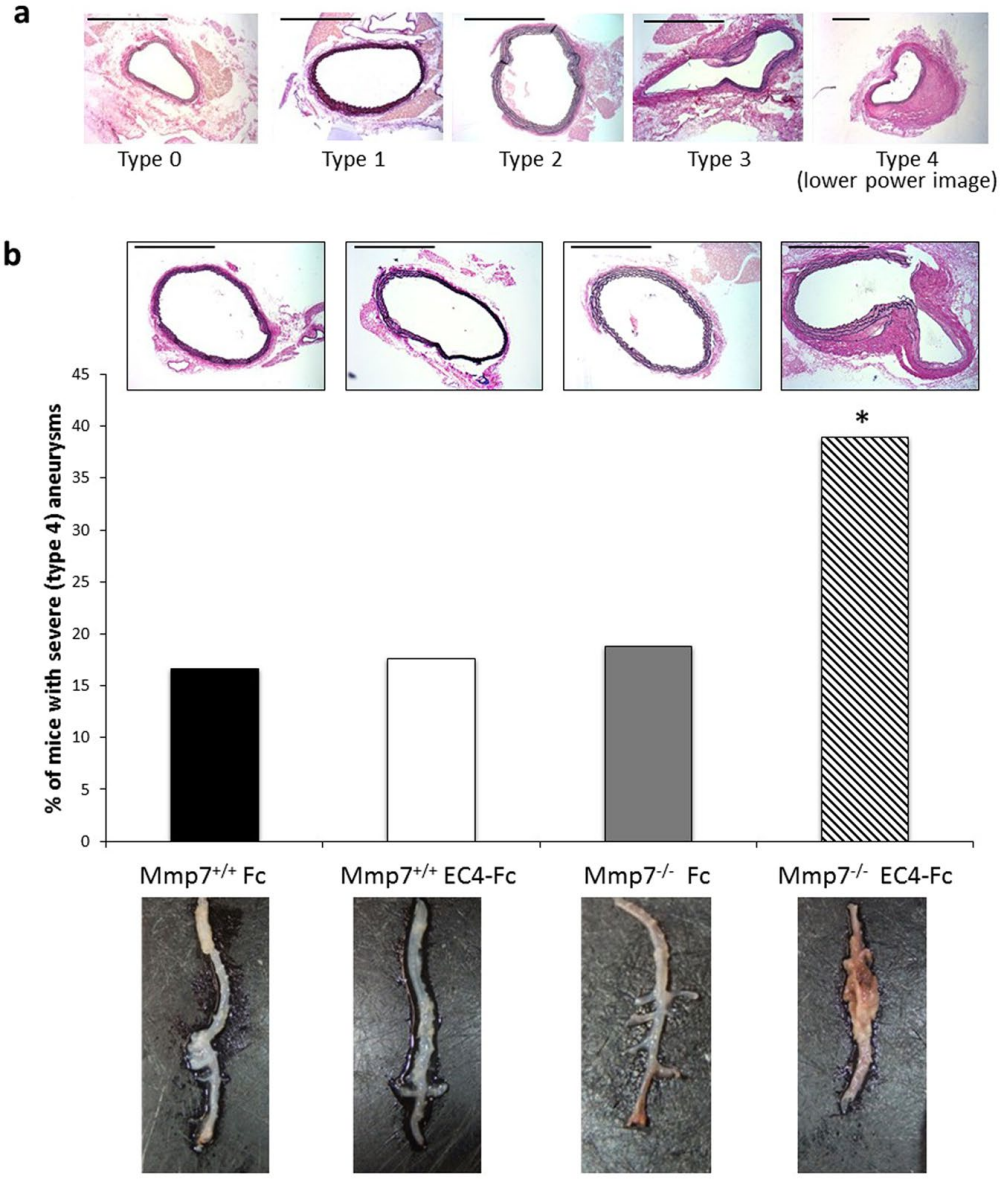
Ang-II induced aneurysm severity in Apoe^−/−^ mice was increased by combined Mmp-7 deletion and EC4-Fc over-expression. (**a**) Elastin van Gieson staining of aneurysms to show the AAA typing system used to rate the severity of the aneurysm. 0: Normal, 1: Dilated, 2: Dilated with atherosclerosis and/or 2 or less elastic lamina breaks, 3: Dilated with atherosclerosis and/or 3 or more elastic lamina breaks, 4: Ruptured. Scale bars indicate 500 µm. (**b**) Representative images of the Elastin Van Gieson (EVG) staining, scale bars indicate 500 µm. Graph shows the percentage of mice with type 4 aneurysms, typed from the EVG stained sections. * indicates a significant difference from WT Fc (n = 17–19, p < 0.05, Fisher’s exact test). Representative macroscopic photographs of the abdominal aortae prior to histological analysis.

Ang-II induced aneurysms were typed according to their severity (Fig. 2a, more detail in methods). Our analysis showed that whilst deletion of Mmp-7 or increased plasma levels of EC4-Fc on their own had no effect on aneurysm severity, in combination, aneurysm severity was significantly increased (type 4: ruptured) (Fig. 2b). This can be seen macroscopically in the representative photographs in Fig. 2b.

**Figure 3:**
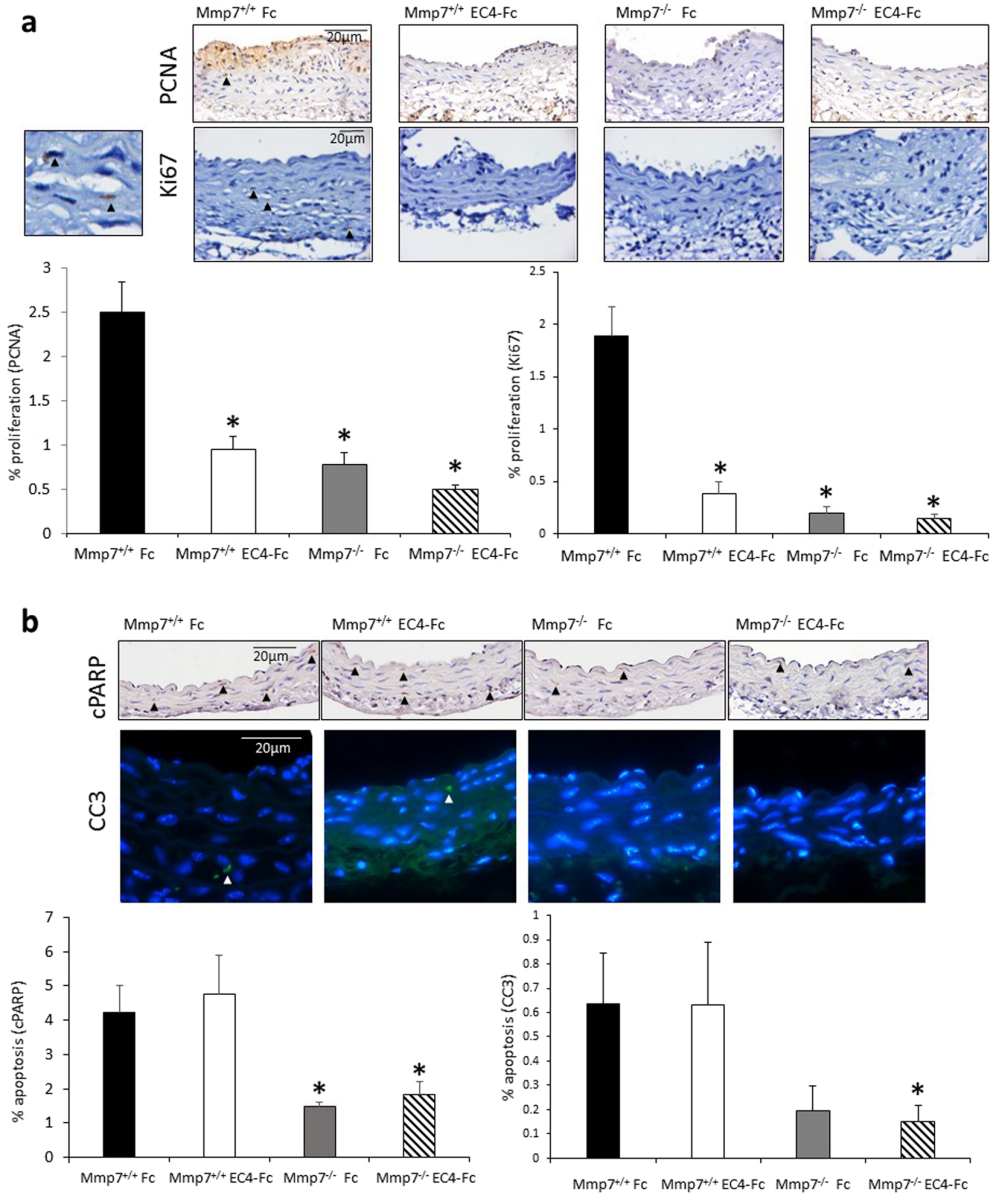
Combined Mmp-7 deletion and EC4-Fc over-expression resulted in reduced vessel wall proliferation and apoptosis in Ang-II induced abdominal aortic aneurysms in Apoe^−/−^ mice. (**a**) Representative images of proliferation markers: PCNA and Ki67 immunohistochemistry in the mouse abdominal aortae. Positive cells are brown, negative nuclei are blue. Arrows indicate some positive cells. Scale bar indicates 20 µm. Small panel (left-handside) shows high power of Ki67 immunohistochemistry with two positive (brown cells). Analysis of immunohistochemistry is shown in the graphs. Proliferation was measured by counting the proportion of positive cells. * indicates a significant difference from Mmp-7^+/+^ Fc. (mean + SEM, n = 17–19, p < 0.05, ANOVA, Student Newman Keuls Post test). (**b**) Representative images of apoptosis markers: cleaved PARP (cPARP) and cleaved caspase-3 (CC3) immunohistochemistry in the mouse abdominal aortae. For cPARP: positive cells are brown, negative nuclei are blue. For CC3: positive cells are green, negative nuclei are blue. Arrows indicate some positive cells. Scale bar indicates 20 µm. Analysis of immunohistochemistry is shown in the graphs. Apoptosis was measured by counting the proportion of positive cells. * indicates a significant difference from Mmp-7^+/+^ Fc (mean + SEM, n = 17–19, p < 0.05, Student Newman Keuls Post test for cPARP, unpaired t test for CC3).

We observed that there was a significant reduction in proliferation (PCNA and Ki67) with both single treatments and the combined treatment (Fig. 3a). There was also a significant reduction in apoptosis (cleaved PARP (cPARP) and cleaved caspase-3) with Mmp-7 deletion and the combined treatments, but not with EC4 alone (Fig. 3b). There were no significant differences in any of the other parameters measured, including smooth muscle cell and macrophage content, cell density, as well as the cellular senescence marker p16 (Table 1, representative images are shown in Supplementary Figure 2).

**Table 1:** Analysis of mouse abdominal aortae using elastin van Gieson staining and immunohistochemistry for actin and GSL.

	Mmp-7^+/+^ Fc (n = 18)	Mmp-7^+/+^ EC4-Fc (n = 17)	Mmp-7^−/−^ Fc (n = 17)	Mmp-7^−/−^ EC4-Fc (n = 19)
**Smooth muscle cell content**				
%: actin	95.9 ± 1.6	92.5 ± 3.3	94.3 ± 1.2	91.2 ± 1.7
%: desmin	77.0 ± 4.4	79.7 ± 3.0	68.0 ± 4.7	81.5 ± 4.4
**Macrophage content**				
%: GSL	0.19 ± 0.07	0.45 ± 0.17	0.41 ± 0.23	0.24 ± 0.12
%: CD68	0.73 ± 0.35	0.61 ± 0.10	0.57 ± 0.15	0.88 ± 0.43
Cellular Senescence (%: p16)	4.01 ± 2.04	2.22 ± 0.59	2.19 ± 0.61	3.42 ± 1.07
Cell density (cells/area)	0.66 ± 0.05	0.65 ± 0.07	0.87 ± 0.13	0.57 ± 0.02
Medial area (µm² × 1000)	252 ± 77	280 ± 98	117 ± 16	164 ± 43
Lumen diameter (µm)	678 ± 37	677 ± 39	649 ± 38	630 ± 41
Medial thickness (µm)	47.2 ± 2.4	45.4 ± 1.9	43.5 ± 2.4	45.9 ± 2.9
Elastic lamina breaks (# broken layers)	2.2 ± 0.4	2.1 ± 0.5	1.9 ± 0.4	2.6 ± 0.5

### EC4-Fc and MMP-7 inhibitor reduced proliferation and apoptosis of human aortic SMCs

**Figure 4:**
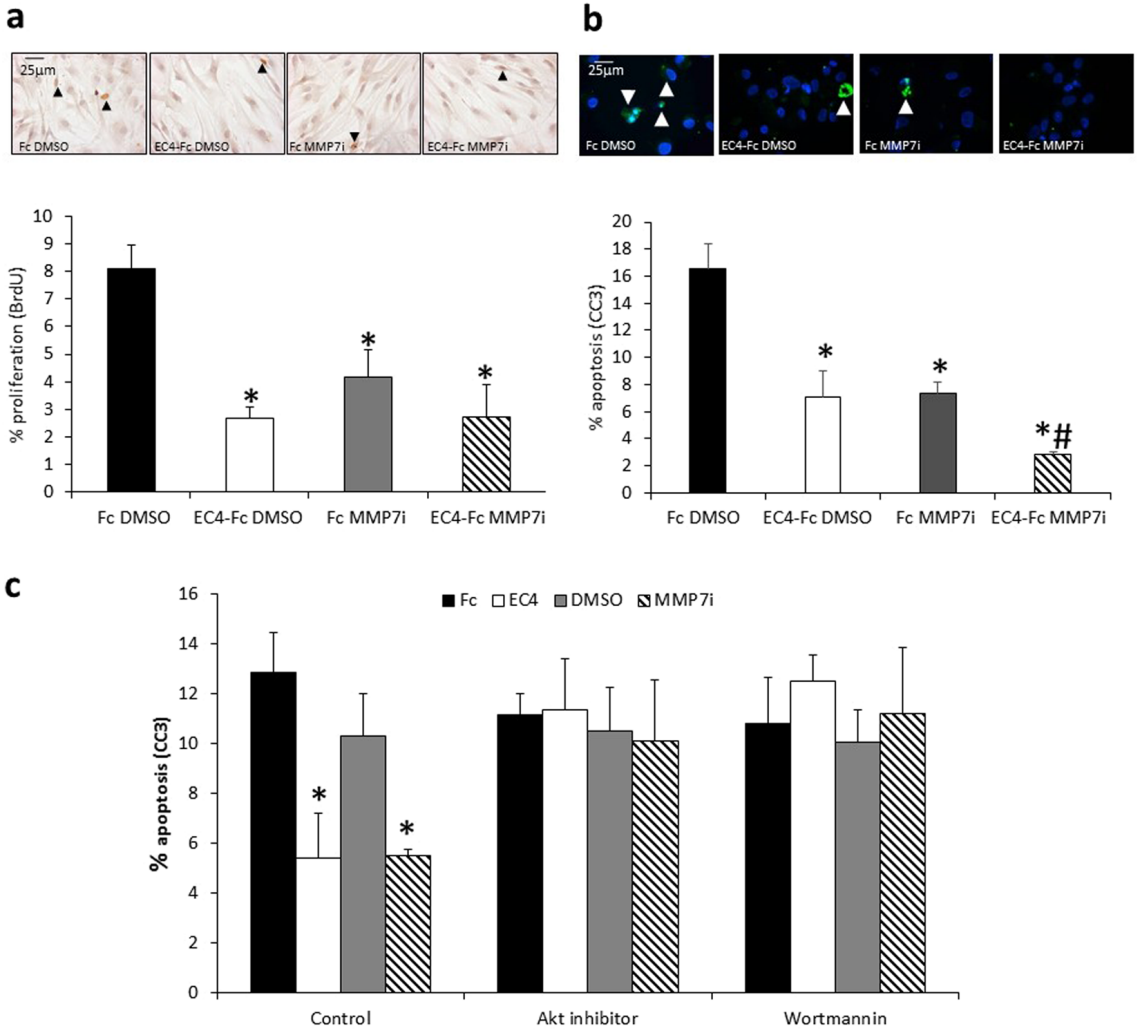
EC4-Fc and MMP-7 inhibitor reduced proliferation and apoptosis of human aortic SMCs. (**a**) Human aortic VSMCs were treated with 20ng/mL PDGF + 20ng/mL bFGF to stimulate proliferation for 24 hours with BrdU, and 100pM Fc or EC4-Fc and 1% DMSO or 0.1 µM MMP-7 inhibitor, then BrdU incorporation quantified. * indicates a significant difference from Fc DMSO (mean + SEM, n = 4, p < 0.05, ANOVA, Student Newman Keuls Post test). Representative images of BrdU immunocytochemistry. Arrow heads indicate some positive cells, in brown. Nuclei are shown in blue. Scale bar indicates 25 µm. (**b**) Human aortic smooth muscle cells were treated with 200ng/mL Fas-L for 24 hours to induce apoptosis, in the presence of 100pM Fc or EC4-Fc and 1% DMSO or 0.1 µM MMP-7 inhibitor. Apoptosis was measured by cleaved caspase-3 (CC3) and the percentage of positive cells was counted. * indicates a significant difference from DMSO Fc. # indicates a significant difference from all other conditions (mean + SEM, n = 4, p < 0.05, ANOVA, Student Newman Keuls Post test). Representative images of cleaved caspase-3 immunocytochemistry. Arrowheads indicate positive cells in green. Nuclei are shown in blue. Scale bar indicates 25 µm. (**c**) Human aortic smooth muscle cells were treated with 200ng/mL Fas-L for 24 hours to induce apoptosis, in the presence or absence of 100pM Fc or EC4-Fc and 1% DMSO or 0.1 µM MMP-7 inhibitor as well as 10 µM Akt inhbitor or 20 nM PI3-kinase inhibitor (Wortmannin). Apoptosis was measured by cleaved caspase-3 (CC3) and the percentage of positive cells was counted. * indicates a significant difference from Control Fc (mean + SEM, n = 4, p < 0.05, ANOVA, Student Newman Keuls Post test).

To investigate the potential mechanism of action we firstly examined the effect of EC4-Fc and MMP-7 inhibition on primary human aortic smooth muscle cell (HASMC) apoptosis, proliferation and migration, using *in vitro* analyses. Both treatments significantly reduced proliferation induced with bFGF and PDGF (Fig. 4a) and 10% FCS (data not shown). EC4-Fc and MMP-7 inhibitor also reduced apoptosis induced with Fas-L in these cells, and had an additive effect on apoptosis (Fig. 4b), suggesting increased efficacy when used in combination. There was however no effect of EC4-Fc and MMP-7 inhibition on HASMC migration (data not shown).

We investigated whether the pro-survival Akt signalling pathway was involved in the observed anti-apoptotic effects using specific inhibitors of Akt and PI3-kinase, and observed that the anti-apoptotic effects were ablated by inhibitors of either Akt or PI3-kinase (Fig. 4c).

## Discussion

Apoptosis is crucially important in aneurysm development. Therefore, we investigated two therapeutic strategies, which we have previously shown to reduce VSMC apoptosis *in vitro* and in the Apoe^−/−^ mouse model of atherosclerosis: EC4-Fc (truncated N-cadherin) over-expression and Mmp-7 deletion. Conversely, VSMC proliferation is known to be activated following vessel wall degradation, and to contribute to repair of the damaged blood vessel wall.

In this study, we demonstrated that full length N-cadherin protein levels were lower whilst N-cadherin fragment levels were increased and MMP-7 activity was higher in the HAAA compared to HA. Additionally, we confirmed that apoptosis was elevated in HAAA compared to HA, as previously reported^2^. We also showed that proliferation was significantly increased in HAAA compared to HA. This confirms previous findings in the ascending thoracic aorta, suggesting that proliferation is activated to repair the damaged vessel^10^. We proposed therefore that MMP-7 activity and associated loss of N-cadherin may be involved in AAA formation and progression.

Although increased levels of apoptosis and proliferation have been previously shown in aneurysm samples, this study is the first to evidence that N-cadherin protein levels are significantly reduced, and MMP-7 activity is significantly increased in human aneurysmal tissue. This is an interesting finding as it suggests an important mechanism by which VSMC apoptosis may be induced in these cells. We have previously demonstrated that MMP-7 activation occurs after exposure to pro-apoptotic factors in VSMCs and results in cleavage of N-cadherin (thus increasing fragment formation), breaking cell-cell contacts and thus promoting apoptosis^17^, as cell-cell contacts are an important survival signal via activation of Akt^19^. During proliferation, MMP-dependent cleavage of N-cadherin results in its shedding from the cell surface, breaking cell-cell contacts and allowing cell division to occur^18^. It appears that this mechanism may be causing proliferation in aneurysmal tissue.

Our *in vivo* study, using the Ang-II mouse model of aneurysm formation, showed approximately 17% of mice infected with control virus developed severe lesions which is similar to that observed in uninfected mice (16.7%, data not shown). This incidence of AAA development is lower than other published studies using this model^6,8^ which may be due to strain differences, as we have used double knockout mice, variations in the high-fat diet purchased or housing differences. Interestingly, combined deletion of Mmp-7 and overexpression of a soluble N-cadherin mimic EC4-Fc (a 50 kDa fragment) resulted in more severe aneurysms. These unexpected findings prompted us to analyse the aneurysms further. We found that although Mmp-7^−/−^ alone and combined with EC4-Fc reduced apoptosis, this coincided with augmented medial proliferation in the abdominal aorta. Following the elastic lamina breakage and vessel dilation that occurs during aneurysm development, VSMCs undergo proliferation to repair the damaged area^10^. Several other studies have also found that factors which reduce VSMC proliferation result in increased aneurysm severity, for example, Emeto *et al*. suggest that an anti-proliferative factor, urocortin-2, is associated with AAA^20^, or Luo *et al*., who found that aberrant expression of microRNA-9 increases intracranial aneurysm development by reducing proliferation^21^. Together these studies and our data suggest a clear association between reduced VSMC proliferation and severe aneurysm formation. We suggest that increased plasma levels of EC4-Fc and Mmp-7^−/−^ prevent the essential repair mechanism of the VSMCs in the AAA, thus, increasing the severity and likelihood of rupture. This lack of proliferation appears to be more important to aneurysm severity than the reduction in apoptosis caused by our treatments.

To corroborate the *in vivo* data, we performed *in vitro* experiments which showed that both singly and in combination, EC4-Fc and an MMP-7 inhibitor reduced human aortic SMC apoptosis and proliferation. Addition of the PI-3 kinase inhibitor and the Akt inhibitor ablated the anti-apoptotic effects of EC4-Fc and the MMP-7 inhibitor, implying that the two treatments separately and in combination inhibit apoptosis through the PI-3 kinase/Akt signalling pathway. However, further analyses would be required to directly demonstrate the signalling pathways responsible for the anti-apoptotic action of EC4-Fc and the MMP-7 inhibitor.

From the literature, and our previous research, we predict that the mechanisms by which EC4 and MMP-7 inhibition reduce proliferation will be independent of one another. Soluble N-cadherin, which contains an FGF-R binding domain, can reduce proliferation by preventing the nuclear translocation of the FGF-R^22^. It is likely that EC4-Fc is acting in the same manner. MMP inhibition (by BB-94, TIMP-1 or -2) has been shown to retard proliferation by preventing N-cadherin cleavage and subsequent shedding from the cell surface^18^. Maintenance of cell-cell contacts is a well-established anti-proliferative mechanism. Together these data suggest that MMP-7 inhibition or deletion of Mmp-7 is likely to reduce proliferation by maintaining cell-cell contacts.

In conclusion, this study emphasises the importance of VSMC proliferation in aneurysm repair. It appears that maintaining VSMC proliferation is equally important as limiting apoptosis in terms of preventing aneurysm progression. Consequently, future studies should focus on the identification of agents that retard VSMC apoptosis whilst sparing or even promoting VSMC proliferation. We hope that the results of this study will help to guide further research into this important area of study.

## Methods

### Human aortic sample preparation

Samples of human abdominal aortic aneurysm (HAAA) were obtained following emergency or planned surgeries to remove large aneurysms. Ethical permission was gained for the protocols used in this study from the local research ethics committee (Research Ethics Committee #11/H0102/3), as well as from the University of Bristol and University Hospital Bristol Review Boards. Patient consent was not required for these samples as in accordance with the Human Tissue Act 2004 in UK, this surplus tissue was deemed to be waste after surgery which does not require consent for anonymised research purposes. The segments of aneurysmal tissue that were received from surgery were heterogeneous in size and some differences in composition were noted. On average, the segments of tissue were approximately 1cm–2cm long and 1 cm wide. These were then divided into 3 pieces: 1 was formalin fixed for histology, 1 was snap frozen for tissue lysates (western blots and MMP7 activity assay) and 1 was snap frozen for frozen sections (*in situ* zymography). The segment for histology was often subdivided as it was still large for processing. From these, we excluded acellular areas, selecting cellular areas with inflammation (macrophages) and evidence of overlying atherosclerosis to achieve a greater homogeneity between samples.

Samples of control undiseased human aorta (HA) were obtained from the hearts donated to the Bristol Coronary BioBank (Research Ethics Committee #08/HO107/48). Consent was obtained from the patient or their family. All human samples were completely anonymous. These samples were formalin fixed and paraffin embedded, or snap frozen. All methods were performed in accordance with the relevant guidelines and regulations.

### *In situ* zymography

*In situ* zymography was performed as described previously^23^. Briefly, 8–10 micron sections of frozen tissue samples were cut in optimal cutting temperature compound (OCT). Sections were incubated overnight in the dark at room temperature with a 20 µg/mL solution of DQ-gelatin (D-12054 Molecular Probes) diluted in zymogram development buffer (161-0766 BioRad), in the presence or absence of 1 µM MMP-7 inhibitor or 20 mM EDTA. After 24 hours, slides were washed in PBS and mounted in Prolong Gold with DAPI (Life Technologies). Gelatinase activity was visualised as green fluorescence.

### MMP activity assay

Tissue samples were lysed in MMP lysis buffer (50 mM Tris pH7.4, 1 mM monothiolglycerol) and MMP-7 activity quantified using a fluorimetric assay. Samples were incubated with 0.14 mg/mL DQ-gelatin fluorescent substrate with and without 0.5 µM MMP-7 inhibitor (444264, Calbiochem) and fluorescence read using a Fluorostar Optima fluorimeter every 30 minutes from 5 hours until peak fluorescence was achieved. MMP-7 activity (i.e. that inhibited by the MMP-7 inhibitor) was compared with that detected from a standard curve of human active recombinant MMP-7 (444270 Calbiochem).

### Western blotting

SDS lysed tissue extracts were subjected to Western blotting as described previously^18^. Blots were incubated overnight at 4 °C with primary antibodies diluted in Starting block (Pierce, Chester, UK). Antibodies were used at the following concentrations: N-cadherin (BD Biosciences: 610920, 1:2,500), β-actin (Sigma, A5316, 1:10,000). Bound antibodies were detected by rabbit anti-mouse or swine anti-rabbit horseradish peroxidase conjugated antibodies (Dako) and enhanced chemiluminescence (Amersham International).

### Immunohistochemistry (human specimens)

Proliferating and apoptotic cells were identified by immunohistochemistry for proliferating cell nuclear antigen (PCNA, abcam 18197, 1 µg/ml) and cleaved caspase-3 (CC3, R&D Systems, AF835, 10 µg/ml), as described previously^14,24^. Smooth muscle cells were identified by immunohistochemistry for alpha smooth muscle cell actin (Sigma, A2547, 3.1 µg/ml). Non-immune immunoglobulin controls were performed for all protocols by substituting the primary antibody with non-immune immunoglobulin of the same species and at the same concentration (Supplementary Figure 3).

### Helper dependent adenovirus (HDAd) production

Plasmids encoding c-myc tagged Fc and EC4-Fc from our previous study^15^ were cloned into helper dependent adenovirus (HDAd) vectors by Professor Lawrence Chan and Kazuhiro Oka at the Baylor College of Medicine, Houston, USA in the same manner as described previously^24^.

### Mouse Ang-II- induced aneurysm model

Mmp-7^−/−^ mice (on a pure C57Bl/6J strain background, obtained from Jax Mice) were crossed with Apoe null mice (C57Bl/8J) to obtain male mice deficient for both Mmp-7 and Apoe (Mmp-7^−/−^Apoe^−/−^). These mice were bred within the Animal Unit of the University of Bristol. Housing, care and all procedures were performed in accordance with the guidelines and regulations of the University of Bristol and the United Kingdom Home Office (PPL # 30/2911) and this study was approved by University of Bristol Review Board. Male 8 week old mice were fed a high-fat rodent diet containing 21% (w/w) fat from lard supplemented with 0.15% (w/w) cholesterol (Special Diets Services, Witham, UK) for 4 weeks. Then Alzet 2004 osmotic mini-pumps were subcutaneously implanted to deliver Ang-II at 1000 ng/min/kg for 28 days to induce aneurysm formation^25^. Two days prior to implanting the osmotic mini-pumps, the HDAds (2.25 × 10^8^ viral particles of virus per mouse in 150 μL of PBS) were administered via tail vein injection as previously^24^, to increase plasma levels of EC4-Fc and Fc. Mice were maintained on high-fat for a further 28 days, then terminated, abdominal aortas removed and paraffin wax embedded. Histological analysis (Elastin Van Gieson staining) for aneurysm size and elastin content and fragmentation were performed.

The four experimental groups were as follows:

Wild type mice transduced with HDAd Fc (Mmp-7^+/+^ Fc) n = 18

Wild type mice transduced with HDAd EC4-Fc (Mmp-7^+/+^ EC4-Fc) n = 17

Mmp-7 depleted mice transduced with HDAd Fc (Mmp-7^−/−^ Fc) n = 17

Mmp-7 depleted mice transduced with HDAd EC4-Fc (Mmp-7^−/−^ EC4-Fc) n = 19

### Aneurysm scoring system

Aneurysm severity was scored using the following scale (see Fig. 3 for example EVG photographs):

0: Normal

1: Dilated

2: Dilated with atherosclerosis and/or 2 or less elastic lamina breaks

3: Dilated with atherosclerosis and/or 3 or more elastic lamina breaks

4: Ruptured

### Quantification of plasma EC4-Fc and lipoprotein levels

Plasma samples were taken when the mice were terminated, 28 days after HDAd administration and levels of EC4-Fc were analysed by Western blotting. To detect EC4-Fc, immunoprecipitation of c-myc tag using the ProFound c-myc tag IP/co-IP kit was performed (Fisher Scientific, Loughborough, UK). Blots were detected with c-myc tag antibody (Cell Signalling, Danvers, USA), and a swine anti-rabbit secondary antibody (Dako, High Wycombe, UK). Plasma lipid profiles were analysed in terminal plasma samples as previously described^26^.

### Immunohistochemistry (mouse specimens)

VSMCs, macrophages and proliferating cells were identified by immunohistochemistry for α-smooth muscle cell actin (Sigma, A2547, 3.1 µg/ml)and desmin (R&D Systems, AF3844, 5 µg/ml), Griffonia Simplicifolia Lectin-1 (GSL, Vector, B-1205, 2.5 µg/ml) and CD68 (LSBio, LS-C343891, 1 µg/ml) and proliferating cell nuclear antigen (PCNA, abcam 18197, 1 µg/ml) and Ki67 (DAKO, M7249, 3.8 ug/ml), respectively, as described previously^14,24^. Cleaved caspase-3 (R&D Systems, AF835, 10 µg/ml) as described previously^14,24^) and cleaved PARP (abcam, ab32064, 4.8 µg/ml) were used to identify apoptotic cells. Briefly, antigen retrieval was performed by boiling in citrate buffer, blocking step was with 5% goat serum, the primary antibody for cleaved PARP was added and incubated overnight at 4 °C. Biotinylated anti-rabbit secondary antibody was utilised, followed by Extravidin-HRP and DAB (3,3′-diaminobenzidine). Nuclei were counterstained with haematoxylin. Immunohistochemistry for a marker of cellular senescence, p16 (Santa Cruz, sc-1661, 2 µg/ml) was performed as described previously^27^. Non-immune immunoglobulin controls were performed for all protocols by substituting the primary antibody with non-immune immunoglobulin of the same species and at the same concentration (Supplementary Figure 3).

### Cell culture

Human aortic VSMCs at passage 4–8 were propagated from explants using our previously described method^18^. Ethical permission was gained for this study from the local research ethics committee (Research Ethics Committee #11/H0102/3) as well as approval from the University of Bristol and NHS Review Boards. Consent was obtained from the patient or patient’s family for the human aortic tissue. Each experiment was carried out with aortic VSMCs from at least 3 different patients. All methods were performed in accordance with the relevant guidelines and regulations.

### Purification of Fc and EC4-Fc proteins

CHO cells were transduced with 50 pfu/cell of RAd Fc or RAd EC4-Fc as described previously^14^. The conditioned media was collected at 66 and 138 hours after transduction/transfection. The conditioned media was pooled and protein purification was achieved with protein A columns (Amersham Biosciences). The protein concentration was determined using the Bradford Protein assay (Sigma) and compared by Western blotting.

### Apoptosis assay

To enable the detection of apoptotic cells by immunocytochemistry, cells were grown on glass coverslips. Human VSMC apoptosis was induced by culturing in serum-free media with 200 ng/mL Fas-L for 24 hours. Cells were supplemented with 100 pM Fc or EC4-Fc as well as 0.1 µM MMP-7 inhibitor (444264, Calbiochem) to assess their anti-apoptotic effects. In some experiments VSMCs were also treated with 20 nM Wortmannin (Calbiochem) or 10 μM Akt inhibitor (Calbiochem) to inhibit PI-3K and Akt signalling, respectively. Apoptosis was assessed by cleaved caspase-3 immunocytochemistry (R&D Systems, AF835, 1 µg/ml) as previously^19^.

### Proliferation assay

Proliferation of aortic VSMCs in the presence of 20 ng/mL platelet-derived growth factor-BB (PDGF-BB) and 20 ng/mL basic fibroblast growth factor (bFGF) was assessed by performing immunocytochemistry for incorporated bromodeoxyuridine (BrdU, Sigma, B8434, 8.6 μg/ml) as previously^28,29^.

### Migration assay

The migration of VSMCs was determined by plating 4 × 10^4^ cells in 24 well plates with 10% (v/v) foetal calf serum and assessed by a scratch wound assay, as described previously^22^.

### Statistics

Results are expressed as mean ± SEM. All data was checked for normal distribution, normally distributed data were analysed by student t-test for comparison of two groups, non-normally distributed data were analysed by Mann Whitney for comparison of two groups and two-way ANOVA with Student Newman Keuls post-test was used for multiple comparisons with more than two groups of normally distributed data. Fisher’s exact test was used for analysis of severity of AAA data. A significant difference was accepted when p < 0.05.

## Electronic supplementary material

Supplementary figures
