# Supplementary material for: Aneurysm Severity is Increased by Combined Mmp-7 Deletion and N-cadherin Mimetic (EC4-Fc) Over-Expression
Source: Sci Rep. 2017 Dec 11;7:17342. doi: 10.1038/s41598-017-17700-8 (PMC5725451; doi:10.1038/s41598-017-17700-8)
Supplement: Supplementary file 1 — Supplementary figures [file 41598_2017_17700_MOESM1_ESM.pdf]

ANEURYSM SEVERITY IS INCREASED BY COMBINED MMP-7 DELETION AND N-CADHERIN MIMETIC (EC4-FC) OVER-EXPRESSION

Cressida A Lyon, Helen Williams, Rosaria Bianco, Steven Simmonds, Bethan A Brown, Kerry S Wadey, Frank Smith, Jason L Johnson, Sarah J George

Supplementary Figure Legends

Supplementary Figure 1

Uncropped Western blot for N-cadherin which was re-probed for the protein loading control (β-actin) of tissue lysates from HAAA and HA. Cropped images of two lanes (lane 3 = HA and lane 7 = HAAA) are shown in figure 1d.
MW: molecular weight marker. Arrows indicate the detected protein bands of full length N-cadherin (FLNC) of 130kDa and the N-cadherin fragment of 30kDa.

Supplementary Figure 2

Representative images of immunohistochemistry in the mouse abdominal aortae for VSMC markers (actin and desmin), macrophage markers (GSL and CD68), and a marker of cellular senescence (p16). For DAB immunohistochemistry: positive cells are brown, negative nuclei are blue. For fluorescence: positive cells are green, negative nuclei are blue. p16 images are shown independently of DAPI to allow easier visualisation of the positive cells. Scale bar indicates 20µm. Analysis of immunohistochemistry is shown in Table 1.

Supplementary Figure 3

Representative images of immunohistochemistry in the mouse abdominal aortae (PCNA, Ki67, cPARP and CC3) and human AAA (actin, CC3 and PCNA). Positive staining and non-immune immunoglobulin controls are shown for comparison. For DAB immunohistochemistry: positive cells are brown, negative nuclei are blue. For fluorescence: positive cells are green, negative nuclei are blue. Arrowheads indicate positive staining.

Supplementary Figure 1

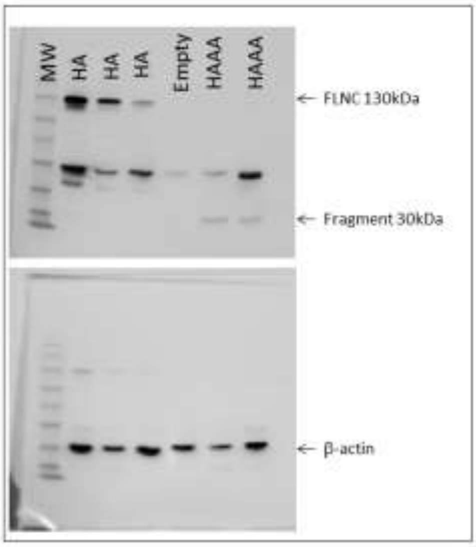

Supplementary Figure 2

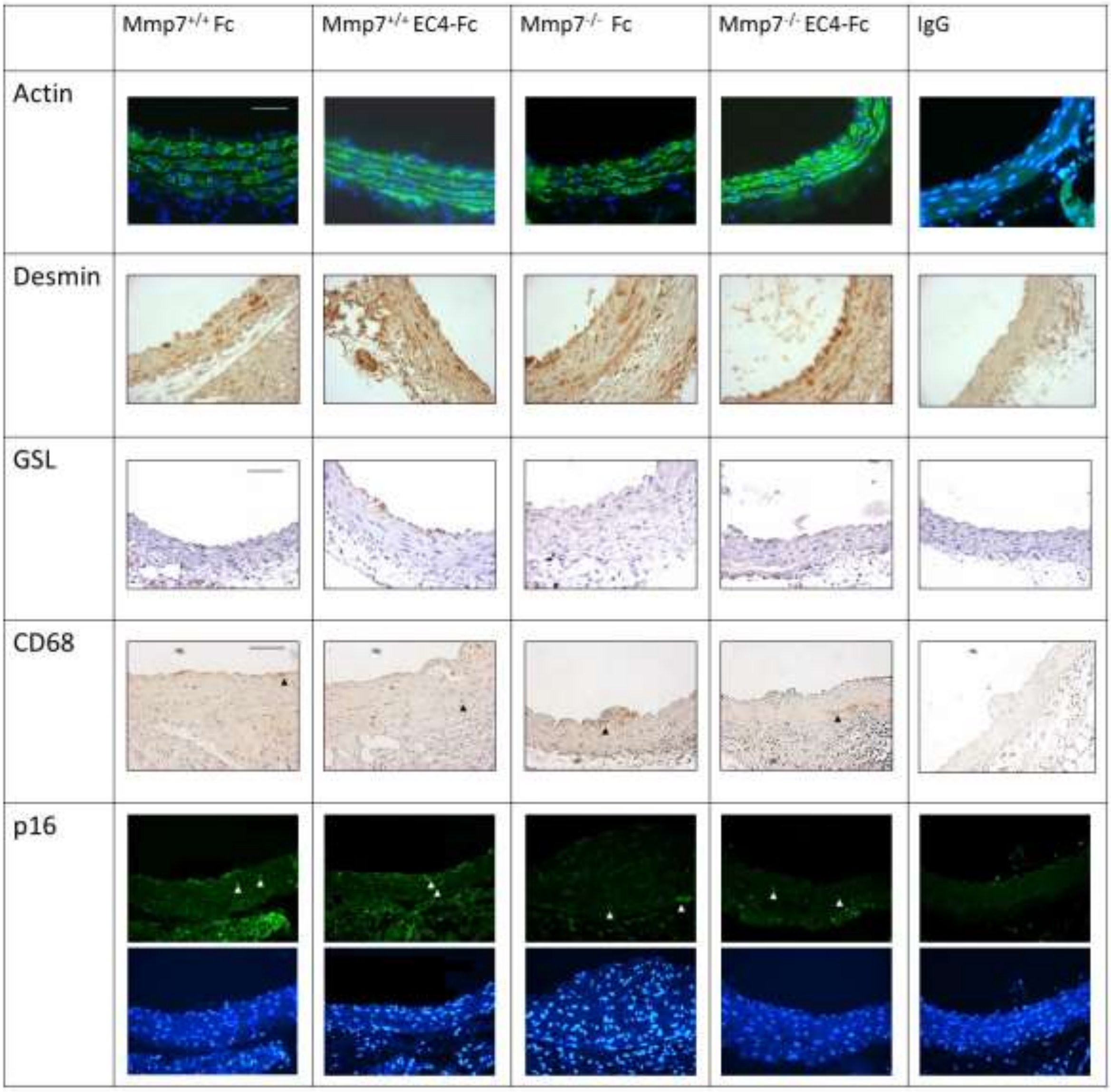

Supplementary Figure 3

Human samples

	Positive staining	IgG negative control
Actin	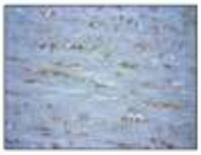	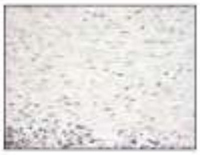
CC3	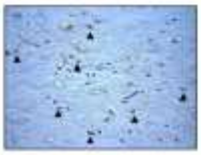	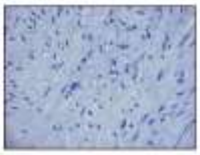
PCNA	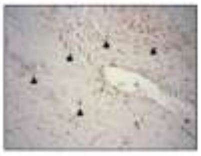	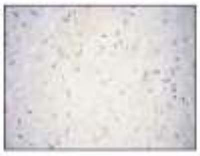

Mouse samples

	Positive staining	IgG negative control
PCNA	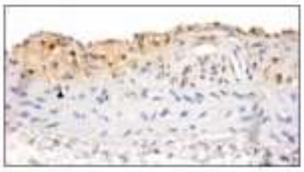	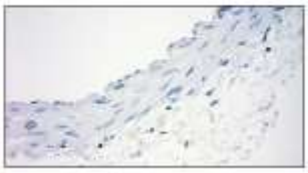
Ki67	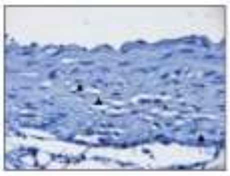	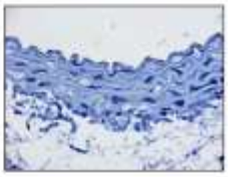
cPARP	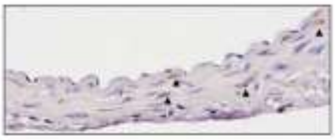	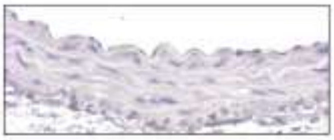
CC3	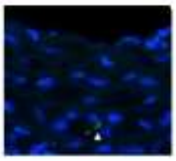	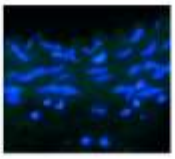
